# Proteomic Study Related to Vascular Connections in Watermelon Scions Grafted onto Bottle-Gourd Rootstock under Different Light Intensities

**DOI:** 10.1371/journal.pone.0120899

**Published:** 2015-03-19

**Authors:** Sowbiya Muneer, Chung Ho Ko, Prabhakaran Soundararajan, Abinaya Manivnnan, Yoo Gyeong Park, Byoung Ryong Jeong

**Affiliations:** 1 Division of Applied Life Science (BK21 Plus), Gyeongsang National University, Jinju, 660–701, Korea; 2 Institute of Agriculture and Life Science, Gyeongsang National University, Jinju, 660–701, Korea; 3 Research Institute of Life Science, Gyeongsang National University, Jinju, 660–701, Korea; Huazhong Agricultural University, CHINA

## Abstract

Although grafting is broadly used in the production of crops, no information is available about the proteins involved in vascular connections between rootstock and scion. Similarly, proteome changes under the light intensities widely used for grafted seedlings are of practical use. The objective of this study was to determine the proteome of vascular connections using watermelon (*Citrullus vulgaris* Schrad.) ‘Sambok Honey’ and ‘Speed’ as the scion and bottle gourd (*Lagenaria siceraria* Stanld.) ‘RS Dongjanggun’ as the rootstock grown under different light intensities (25, 50, 75 and 100 μmol m^−2^ s^−1^). Our proteomic analysis revealed 24 and 27 differentially expressed proteins in ‘Sambok Honey’ and ‘Speed’, respectively, under different light intensities. The identified proteins were largely involved in ion binding, amino acid metabolism, transcriptional regulation and defense response. The enhancement of ion-binding, transcriptional regulation, amino acid metabolism, and defense response proteins suggests a strengthening of the connection between the rootstock and scion under high light intensity. Indeed, the accumulation of key enzymes in the biological processes described above appears to play an important role in the vascular connections of grafted seedlings. Moreover, it appears that 100 μmol m^−2^ s^−1^ results in better protein expression responses in grafted seedlings.

## Introduction

Grafting is widely used in the production of fruit and vegetable crops [1–2] to benefit soil-grown plants with regard to biotic and abiotic stress tolerance for enhanced development [3–4]. In horticulture, grafting is largely used to facilitate crop growth in soil infected with possible soil-borne pathogens [5–6]. A successful grafting is a complex biochemical and structural process that starts with the union of two organisms, followed by callus development and the formation of a functional vascular system [7]. The graft union development is a sophisticated process due to which histological and physiological variations such as initiation and development of organ regeneration are perceived, indeed the exchange of genetic material between different cells of rootstock and scion can also be ensued [8–9]. The morphological and vascular connections using numerous imaging techniques and common histological methods have been extensively studied in several graft union plants [9–12]. At the molecular levels, several recent studies have reported important factors underlying graft union formation [13–16] and have revealed essential genes related to plant hormones, cell cycle, metabolism and signal transduction. The morphological, physiological and few molecular changes during graft union formation have been well documented [10–11]. However, it remains unclear how the two organisms can share a vascular connection after successful grafting. It is possible that a mechanism revealed at the proteome level can provide key information about the proteins/genes involved in the vascular connections of grafted plants.

The fundamental aspect of plant growth is light, which act as an energy source for photosynthesis and an environmental signal which regard to its intensity, wavelength and direction [17]. Light signals also play a key role in regulating plant hormone signaling mechanisms, such as changes in auxin, abscisic acid (ABA), and gibberellic acid (GA) [18–20]. Similarly, at practical point of view the grafted plants also require environmental control especially light quality and intensity during healing and acclimatization process [21–22] to increase chances for vascular bundles of rootstock and scion to come in contact. Light emitting diodes (LEDs) are being used as an efficient source of light for growing high quality of plants in a closed plant growth chambers. Light emitting diodes are suitable for small mass, safety and durability for better growth and development of plants [17, 23–25]. Indeed it has been observed that light emitting diodes (LEDs) mostly red, blue and white have a good special characteristic and spectral width that can match light quality needed for successful grafting [21–22]. However, a major challenge for successful grafting is also supplying an efficient quantity and quality of light. A possible combinations of different light emitting diodes (LEDs) particularly combination of red, blue and white (R:B:W) at different light intensities can reveal an optimal and suitable light intensity for effective grafting.

Although grafting is largely used in the production of vegetable and fruit-bearing crops for adaptation to biotic and abiotic stress, no information is available about the proteins involved in vascular connections between the rootstock and scion when two different species are grafted together. However, proteomic-based studies can significantly help to reveal the possible relationships between rootstock and scion vascular connections. Similarly, it is also important to determine the changes that occur with the light intensities widely used for growing grafted plants and determine optimal light intensity for practical purposes. Thus, the objective of this study was to determine the proteome of vascular connections (the connected portion of the rootstock and scion) using watermelon (*Citrullus vulgaris* Schrad.) ‘Sambok Honey’ and ‘Speed’ as the scion and bottle gourd (*Lagenaria siceraria* Stanld.) ‘RS Dongjanggun’ as the rootstock under different light intensities (25, 50, 75 and 100 μmol m^−2^ s^−1^ PPFD).

## Material and Methods

### Plant materials, grafting procedure and treatments

The commercial available watermelon (*Citrullus vulgaris* Schrad.) ‘Sambok Honey’ and ‘Speed’ as the scion and bottle gourd (*Lagenaria siceraria* Stanld.) ‘RS Dongjanggun’ as rootstock seedlings were collected from Chojeon Nursery Farm, Gyeongsangnamdo Jinju-city Geumsan-Myeon Jungcheon-ri 507, Korea (a private farm which commercially sells grafted plants and no specific permissions are needed for collection of plants). The two scion genotypes grafted on rootstocks were selected as these two cultivars are extensively used for grafting purpose in Korea because of highest rate of germination and fruit quality. Grafting process was implemented by sharp dissecting blade seven days post-germination. Seedlings were selected with uniform length and was cut transversely. The rootstock was cut close to the shoot apical meristem and scion was cut intermediate from the base of the hypocotyl. The forceps were used to retain the seedlings firm while cutting. The wounding were made swiftly to keep the lesions clean and smooth. The scion was up-lifted to bring it to the rootstock, and the rootstock was carefully picked up to approach the scions and connect them together. The rootstock and scion was carefully adjusted at the graft junctions and was carefully sealed with a grafting clips. The grafted plants were grown under combination of white:red:blue light emitting diodes (LEDs) at different photon flux densities of (25, 50, 75 and 100 μmol m^−2^ s^−1^ PPFD). The different photon flux density was measured using a portable photo/radiometer HD-2102.2 (Delta, OHM, Padova, Italy) and was separately controlled by adjusting both electric currents and number of light bulbs for the LEDs. All treatments were done in a culture room, employing separate plots for the different light intensities. The room was ventilated to maintain the CO_2_ level the same as that of the outside atmosphere. The relative humidity was maintained at 98±2% with a 12 h photoperiod and a temperature of 23°C during the light and dark period. After two weeks of grafting grown under different light intensities graft union (vascular portions) were excised smoothly 2–3 cm around the grafted junction and immediately frozen in liquid N_2_ and stored in a deep-freezer (-80°C) for further analysis. For physiological measurements the grafted plants were observed 4 days after grafting up to 2 weeks. The time point for physiological observations (4 d-2 weeks) were selected as physiological changes occur after 24–48 h prior to morphological changes such as formation of brownish layers. Proteomic study in graft union was studied 2 weeks after grafting since callus formation occurs after 2–3 weeks prior to grafting and exchange of proteins and genetic material between graft unions are more available at this point.

### Measurement of biomass and hardness of vascular connections

The biomass of rootstock and scion (around graft union) harvested from 4 days up to 2 weeks were constantly weighed on weighing balance and were recorded respectively. The hardness of rootstock and scion (around graft union) were observed by pressure tester (DFT-01, Proem, Italy).

### Protein sample preparation

The vascular (connected portion between rootstock and scion) portion of rootstock and scion were smoothly cut down 2–3 cm 2 weeks during healing process after grafting with the help of sharp razor and were homogenised in liquid nitrogen in precooled pestle and mortar. The proteins were extracted in commercially available protein extraction buffer kit (Bio-Rad, Hercules, CA, USA) according to manufacturer’s instructions. About 2–3 ml of extraction buffer contacting 8M urea, 4% CHAPS, 40 mM Tris, 0.2% (w/v) bio-lyte (*pI* 3–10) were added to 100 mg of powdered frozen samples. The samples were vortexed and placed on ice, then sonicated with an ultrasonic probe to disrupt the cells and fragments of the genomic DNA (S1 Fig.). The sonicated samples were centrifuged at maximum speed in a microcetrifuge for 20 min at 4°C to pellet down the cell debris. The resulting supernatant were transferred to a clean e-tubes and extracted protein samples in the form of supernatant was quantified by Bradford using BSA as a standard curve.

### Two-dimensional gel electrophoresis (2-DE) and staining

For isoelectric focussing (IEF), the Multiphor II system (GE Healthcare) and IPG strip (pH 4–7, nonlinear, 11 cm, GE Healthcare) were used according to manufacturer’s instructions with minor modifications. To minimize experimental errors, extracted protein samples from three biological replicates were subjected to focussing at the same time. The dry IPG strips were rehydrated for 12 h in 250 μl rehydration buffer (Bio-Rad, Hercules, CA, USA) containing 70 μg of protein. Focussing was performed at 20°C at a current limit of 50 μA per IPG strip at 20°C in four steps: 200 V for 0:01 (h:min), 3500 V for 1:00 (h:min), 3500 V for 1:30 (h:min) and a final step 3500 V for 1–2 h until the final volt reaches to 10000 Vh. The gel strips were then equilibrated by incubating on a shaker in 2–3 ml of an equilibration buffer 1 for 30 min [8M urea, 2% SDS, 50 mM Tris-HCl (pH 8.8) 20% (v/v) glycerol, 1% DTT] followed by 2–3 ml of an equilibration buffer 2 for 30 min [same content as equilibration buffer 1 except DTT was replaced by 2.5% iodoacetamide]. The second dimension was performed on 12.5% (w/v) SDS-polyacrylamide gels on PROTEAN II (Bio-Rad, Hercules, CA, USA) with a constant voltage of 70 V for 4–5 h until the run was complete. For molecular weight of proteins, commercial pre-stained molecular marker (Intron Biotechnology, Seongnam-City, South Korea) were run on one side of the SDS-PAGE gels. Protein spots were visualized by staining 2D gels with silver stain. The stained gels were scanned using a high resolution scanner (EPSON) and gel images were analyzed using PD-Quest basic software (Bio-Rad, Hercules, CA, USA). The 2D gels were stored in 1% acetic acid until further analysis.

### Image and data analysis

In each treatment, three independent biological replicate plants were taken. Gels were taken under constant settings by a photo imager. The protein spots from all 2-DE gels were matched to have the same number across all gels. A master gel image containing matched spots across all gels was auto-generated. The missing spots from the 2-DE gels were resolved using an extensive analysis ‘landmark’ tool and respective spot volumes were normalized according to the total gel image density as recommended by the PD-Quest software (version 7.2.0; Bio-Rad, Hercules, CA, USA). The gels from all the treatments were compared by creating three different replicate groups, and each replicate group contained the gel images corresponding to a specific treatment. In each group, an average quantity was determined for each spot, and pairwise quantitative and statistical analysis sets were generated by comparing the volume of a given spot across all treatments. The statistical significance of the quantitative data was determined by student’s *t*-test (n = 3, *p* <0.05) at a 95% confidence level. Where the identified proteins showed a 1.5-fold or more change in the amount among all treatments.

### Protein in gel digestion

The differential protein spots were excised manually from the 2D gels with the help of a clean razor blade and were chopped into small pieces. The excised spots were transferred to 0.5 ml clean microfuge tubes. The gel pieces were destained with freshly-prepared 30 μl of a 1:1 (v/v) mixture of the two destained reagents K_3_[Fe(CN)_6_] (potassium ferricyanide) and Na_2_S_2_O_3_ (sodium thiosulphate pentahydrate) by incubating for 30 min at room temperature with gentle agitation. The destaining solution was removed and gel particles were washed with distill water and 50 mM NH_4_HCO_3_/ACN (v/v) for 15 min. The gel particles were then covered again with ACN for 2–5 min. The gel particles were then dried in a vacuum centrifuge. After drying the gel particles were rehydrated in 10 mM dithiotreiotol/50 mM NH_4_HCO_3_ by incubating at 56°C for 45 min. The e-tubes containing gel particles were cooled to room temperature in dark conditions and a rehydrated solution was removed. The gel particles were again washed with 50 mM NH_4_HCO_3_ and ACN with one or two changes for 15 min per change. The gel particles were covered with ACN to shrink the gel pieces. The gel particles were dried in a vacuum centrifuge. After washing steps, the gel particles were treated with freshly prepared 5 ng of trypsin (Sigma Chemical Co., St Louis, MO, USA) prepared in 1 M HCl to cover the gel and was incubated at 37°C for 30 min. After incubation the excess enzyme was removed carefully and approximately 2–3 μl 25 mM of NH_4_HCO_3_ was added to keep gel wet overnight at 37°C. After overnight incubation the microfuge tubes containing gel particles were spun down and resulting supernatants (peptide mixtures) were collected in new microfuge tubes. The resulting peptides were vacuum dried and dried peptides were dissolved in a 3–5 μl of sample solution containing 50% ACN and 0.1% trifluroacetic acid (TFA). The solutions were stored at-20°C until further use.

### Protein identification using MALDI-TOF MS and MALDI-TOF/TOF MS

The digested peptide solution (in 50% ACN, 0.1% TFA) was spotted onto the MALDI-TOF/TOF target plate with a pipette. MALDI-MS analysis was performed with a Voyager DE-STR mass spectrometer (Applied Biosystems, Framingham, MA, USA). The peptide fragmentation spectra were detected and collected in the reflection/delayed mode. The data were analyzed with MoverZ (http://www.proteomics.com) and the MASCOT database (http://www.matrixscience.com). All MALDI-TOF MS spectra were searched against the National Center for Biotechnology Information (NCBI) protein database (version 20131215; 35099569 sequences). Single missed cleavage, oxidation of methionine, and cysteine modified by iodoacetamide were allowed for database searching for protein identification. An ABI 4800 Plus TOF–TOF Mass Spectrometer (Applied Biosystems, Framingham, MA, USA) was employed for MS and MS/MS analyses of the peptides. The instrument was set at 200 Hz ND: 355 nm YAG laser operations. Signal/noise ratios > 25 and the ten with higher intense ions were used to following MS/MS analysis in 1 kV mode, 1000–1250 consecutive laser exposure. The MS and MS/MS spectra data were analyzed using NCBI and Protein Pilot V.3.0 database software (with the MASCOT V.2.3.02 database search engine) at 50 ppm of mass tolerance. Single missing pick, oxidation of methionines, and carbamidomethylation of cysteines were allowed for the MS/MS spectra search in the databases. Individual peptide ions scores were searched using a statistically significant threshold value of *p* = 0.05.

### Protein functional classifications

The identified proteins were classified into different categories of biological processes in which they are involved according to gene ontology (http://www.geneontology.org/).

### Vascular staining (absorbable flower dye blue staining)

Vascular staining method was followed as described previously by Olmstead et al. [11]. Samples were fixed in 0.1% absorbable flower dye blue for 20–30 min and were rinsed with water. The rootstock and scion samples were then cut into transverse and longitudinal section with the help of sharp surgical razor/blade and were observed under light microscope (Nikon Eclipse Ci-S/Ci-L, Japan)

## Results and Discussion

### Physiological response

**Fig 1:**
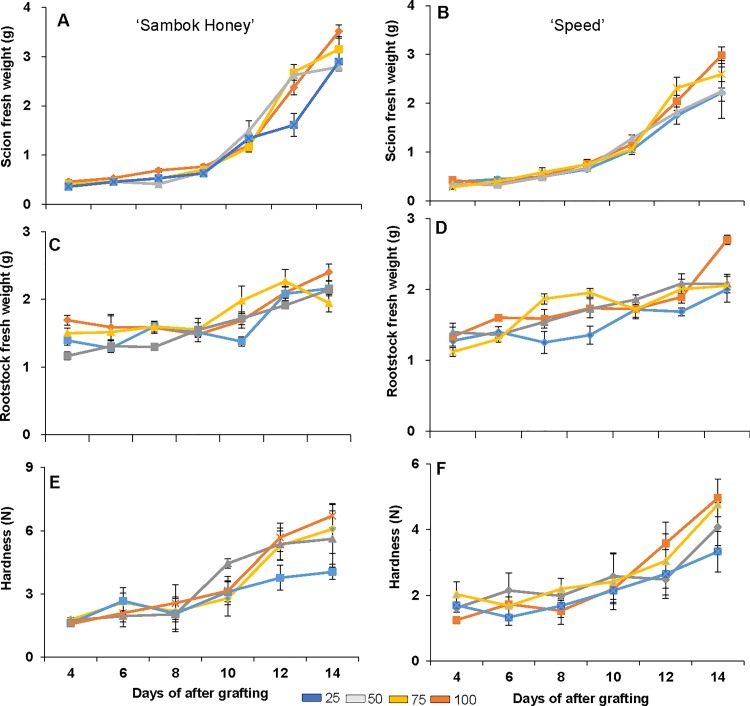
Scion/ rootstock fresh weight and hardness in watermelon (*Citrullus vulgaris* Schrad.) ‘Sambok Honey’ and ‘Speed’ as the scion and bottle gourd (*Lagenaria siceraria* Stanld.) ‘RS Dongjanggun’ as rootstock seedlings grown under different photon flux densities (25, 50, 75 and 100 μmol m−2 s−1 PPFD). (A-C) scion/rootstock fresh weight of ‘Sambok-Honey’ (B-D) scion/ rootstock fresh weight of ‘Speed’ (E) hardness of ‘Sambok-Honey’ (F) hardness of ‘Speed’.

The physiological responses in vascular connections were studied to select optimal time point for proteomic analysis after grafting under different light intensities. Moreover, physiological responses were studied to support the hypothesis that 100 μmol m^-2^ s^-1^ are optimal light intensity for better plant growth and development after grafting. The physiological responses such as biomass of scion and rootstock (grafted portion/vascular connection) (Fig. 1A, 1B, 1C and 1D) showed highest values at 100 μmol m^-2^ s^-1^ compare to other light intensities two weeks after grafting. Furthermore, the graft union pressure tester showed highest hardness of graft union at 100 μmol m^-2^ s^-1^ and lowest at 25 μmol m^-2^ s^-1^ light intensity (Fig. 1E and 1F) two weeks after grafting. Generally the non-grafted plants (normal) have also previously shown that 100–200 μmol m^-2^ s^-1^ light intensity are optimal for increased biomass of plants such as in *Lactuca sativa* [17] and *Cassia angustifolia* [26]. Therefore, our results supported the hypothesis that higher light intensity (100 μmol m^-2^ s^-1^ as shown by our results) might be an optimal light intensity for better graft union formation in watermelon (*C*. *vulgaris* Schrad.) ‘Sambok Honey’ and ‘Speed’ as the scion and bottle gourd (*L*. *siceraria* Stanld.) ‘RS Dongjanggun’ as the rootstock.

### Two-dimensional (2-DE) protein profile in the rootstock and scion vascular connection

**Fig 2:**
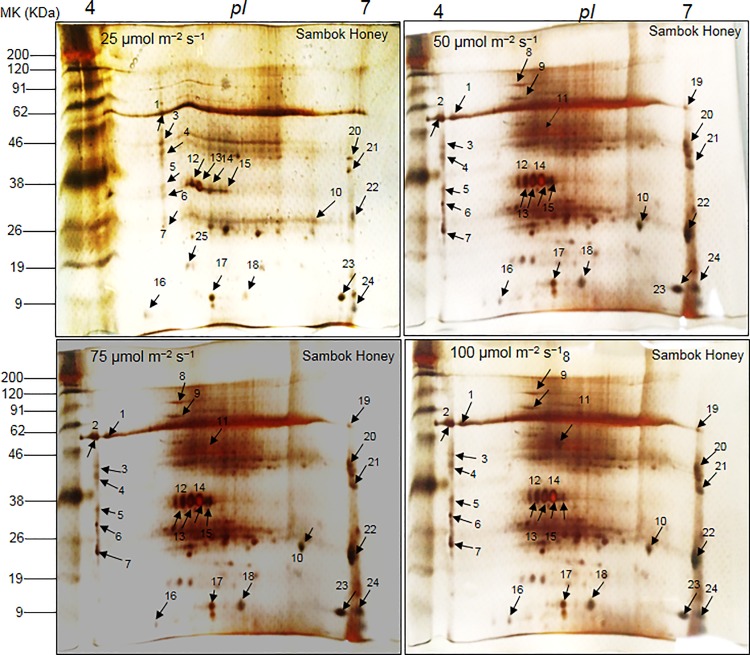
2-DE reference maps of vascular connections (connected portions) in watermelon (Citrullus vulgaris Schrad.) ‘Sambok Honey’ as the scion and bottle gourd (*Lagenaria siceraria* Stanld.) ‘RS Dongjanggun’ as rootstock seedlings grown under different photon flux densities (25, 50, 75 and 100 μmol m−2 s−1 PPFD). Proteins from vascular connections (70 μg) were elctrofocused on a pH 4–7 IPG strip (11 cm), separated onto 12.5% (w/v) SDS-PAGE. The gels were silver stained and visualized as described in experimental section. Protein spots marked by arrows were identified by MALDI-TOF/TOF-MS as described in experimental sections.

**Fig 3:**
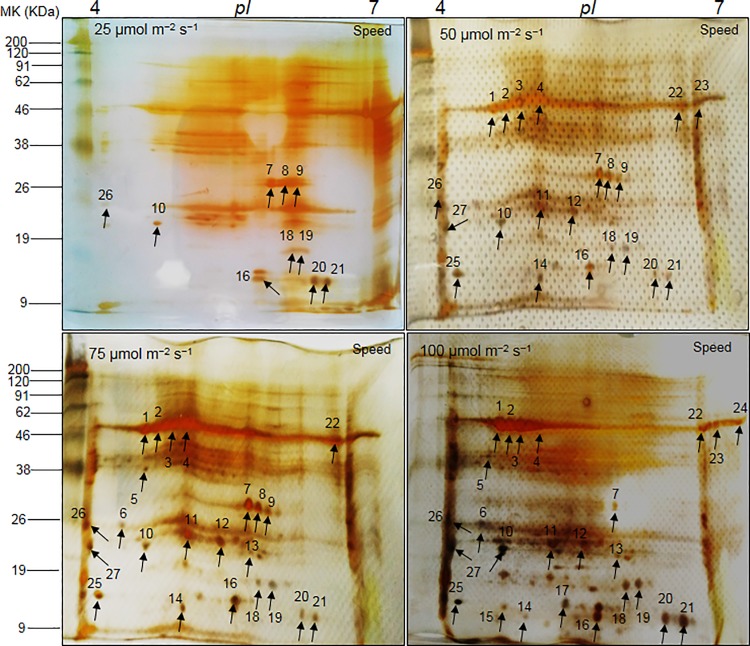
2-DE reference maps of vascular connections (connected portions) in watermelon (*Citrullus vulgaris* Schrad.) ‘Speed’ as the scion and bottle gourd (*Lagenaria siceraria* Stanld.) ‘RS Dongjanggun’ as rootstock seedlings grown under different photon flux densities (25, 50, 75 and 100 μmol m−2 s−1 PPFD). Proteins from vascular connections (70 μg) were elctrofocused on a pH 4–7 IPG strip (11 cm), separated onto 12.5% (w/v) SDS-PAGE. The gels were silver stained and visualized as described in experimental section. Protein spots marked by arrows were identified by MALDI-TOF/TOF-MS as described in experimental sections.

The protein profile distribution patterns in 2-DE images from the grafted portions of watermelon (*C*. *vulgaris* Schrad.) ‘Sambok Honey’ and ‘Speed’ as the scion and bottle gourd (*L*. *siceraria* Stanld.) ‘RS Dongjanggun’ as the rootstock grown under different photon flux densities (25, 50, 75 and 100 μmol m^−2^ s^−1^ PPFD) showed considerable variations (Fig. 2 and Fig. 3). According to a detailed comparison of 2D images analyzed by PD-Quest software (Bio-Rad, Hercules, CA USA), at least 455 protein spots were detected on each gel. Among the 455 protein spots, 24 proteins from ‘Sambok Honey’ and 27 from ‘Speed’ were differentially expressed under the different intensities of light. It was observed that under 25 μmol m^-2^ s^-1^, the protein spots were mostly down-regulated or absent compared to the other light intensities (50 and 75 μmol m^-2^ s^-1^). Highly up-regulated proteins were observed under 100 μmol m^-2^ s^-1^.

A gel-based analysis showed that protein spots were highly down-regulated under 25 μmol m^-2^ s^-1^ light intensity, they were highly up-regulated under 100 μmol m^-2^ s^-1^. The down-regulation of protein spots under 25 μmol m^-2^ s^-1^ showed a progressive depletion of proteins involved in signaling and amino acid biosynthesis, which are important processes for protein synthesis [27–28]. A progressive decrease in protein synthesis under a low light intensity (25 μmol m^-2^ s^-1^) also suggests that a limited amount of light may be associated with the production of reactive oxygen species (ROS) in the vessels (xylem and phloem) of grafted plants during photosynthesis and transpiration, leading to incorrect protein folding or protein degradation. A reduction in protein synthesis has been observed in several plants [29–31] under abiotic stress. Indeed, protein degradation was previously observed in bottle gourd rootstock-grafted watermelon seedlings [4] under salt stress. Although grafting is being used to protect crop plants from several biotic/abiotic stresses, it is still unclear which light intensity may be optimal for the healing of the mechanical injury suffered during the process of grafting. Based on our results, we propose that 100 μmol m^-2^ s^-1^ can be used as an optimal light intensity to prevent protein loss, especially proteins involved in rootstock-scion vascular connections.

### Protein identification and functional classification by MALDI/TOF/TOF-MS

The differentially expressed proteins related to vascular connections (connected grafted portion) between watermelon scions grafted onto bottle gourd rootstock under different light intensities (25, 50, 75 and 100 μmol m^-2^ s^-1^) were then identified. Twenty-four ‘Sambok Honey’ protein spots differentially expressed under different light intensities were analyzed by mass spectrometry (MALDI-TOF/TOF-MS) (Table 1). The seven up-regulated proteins under 100 μmol m^-2^ s^-1^ are related to ion binding and identified as Putative DAK2 domain-containing protein (spot 11), RNA recognition motif (spot 17), Receptor kinase LRK10 (spot 18), Maturase K (19), 50S ribosomal protein L2 (spot 20), and Alanine-glyoxylate aminotransferase (spots 21 and 22).

**Table 1:** Proteins identified by MALDI-TOF/TOF-MS in watermelon ‘Sambok Honey’ as the scion and bottle gourd ‘RS Dongjanggun’ as rootstock.

Spot No.	NCBI accession number	Protein Name	Biological function	Plant Species	M*r* Value	Calculated p*I*/Exp p*I*	MASCOT Score	Sequence coverage (%)
1	F4IJ86	Homeobox-leucine zipper protein ATHB-7	Transcriptional regulation	*Arabidopsis thaliana*	30421	5.52/4.6	47	20
2	A9PK54	Transcription elongation factor SPT4 homolog	Transcriptional regulation	*Populus trichocarpa*	13606	5.66/4.0	44	56
3	M0UZT2	Beta-galactosidase	Carbohydrate metabolism	*Hordeum vulgare*	81149	5.66/4.0	42	50
4	-	-	-	-	-	-	-	-
5	V4KH47	ATP-dependent Clp protease proteolytic subunit	Amino acid biosynthesis	*Eutrema salsugineum*	42995	8.85/4.0	64	41
6	E1ZEF2	Cysteine synthase	Amino acid biosynthesis	*Chlorella variabilis*	36901	8.79/4.0	52	39
7	O48573	Disease resistance protein-	Defense response	*Arabidopsis thaliana*	135194	5.75/4.0	66	14
8	D8RG99	Inner arm dynein 3–2	Microtubule based movement	*Selaginella moellendorffii*	368702	5.44/5.44	67	11
9	-	-	-	-	-	-	-	-
10	V4K6A2	Uncharacterized protein	-	*Eutrema salsugineum*	77378	5.1/5.4	77	24
11	Q00YY4	Putative DAK2 domain containing protein	Ion binding	*Ostreococcus tauri*	88997	6.69/6.6	83	18
12	D8R7J6	Putative uncharacterized protein	-	*Selaginella moellendorffii*	99451	8.72/5.5	77	21
13	D8RG99	Inner arm dynein 3–2	Microtubule based movement	*Selaginella moellendorffii*	368702	5.44/5.6	70	9
14	A2YMB7	Beta-amylase	Carbohydrate metabolism	*Oryza sativa*	55464	5.3/5.7	59	34
15	E0CSS7	Putative uncharacterized protein	-	*Vitis vinifera*	135140	8.57/6.0	70	14
16	D7KEC0	Galactosyltransferase family protein	Protein glycosylation	*Arabidopsis thaliana*	46825	5.9/5.4	61	55
17	Q2A9U7	RNA recognition motif	Nucleotide binding	*Brassica oleracea*	45703	6.5/6.2	54	28
18	Q84K89	Receptor kinase LRK10	ATP binding	*Avena sativa*	72721	7.1/6.4	59	21
19	L7PBV6	Maturase K	RNA binding	*Derris submontana*	61607	9.26/7.0	58	20
20	G7J106	50S ribosomal protein L2	RNA binding	*Medicago truncatula*	15106	11.6/7.0	56	49
21	R4L8I2	Alanine-glyoxylate aminotransferase	Pyridoxal phosphate binding	*Peperomia prostrata*	31935	9.7/7.0	56	20
22	R4L8I2	Alanine-glyoxylate aminotransferase	Pyridoxal phosphate binding	*Peperomia prostrata*	31935	9.7/7.1	56	20
23	M5X1H0	ATP-dependent Clp protease proteolytic subunit	Amino acid biosynthesis	*Prunus persica*	32028	5.9/7.0	45	42
24	K4CH47	Uncharacterized protein	-	*Solanum lycopersicum*	10546	10.0/7.0	57	70

The other 3 proteins are related to amino acid biosynthesis and identified as ATP-dependent Clp protease proteolytic subunit (spots 5 and 23) and cysteine synthase (spot 6). Another important protein was found to be related to defense response and was identified as disease resistance protein (spot 7). In addition to proteins related to translocation (predicted as translocation of nutrient material through phloem), proteins related to carbohydrate metabolism, beta-galactosidase (spot 3) and beta-amylase (spot 14), were also identified. Another important protein in vascular connections was found to be related to the movement of tubules, Inner arm dynein 3–2 (spots 8 and 13)

Moreover, some of the proteins identified are related to signaling and secondary metabolism: Homeobox-leucine zipper protein ATHB-7 (spot 1) and transcription elongation factor SPT4 homolog (spot 2), and Galactosyltransferase family protein (spot 16). Additionally, some proteins were not identified and were listed under the category of unidentified proteins and uncharacterized proteins (spots 4, 9, 10, 12, 15, and 24).

**Fig 4:**
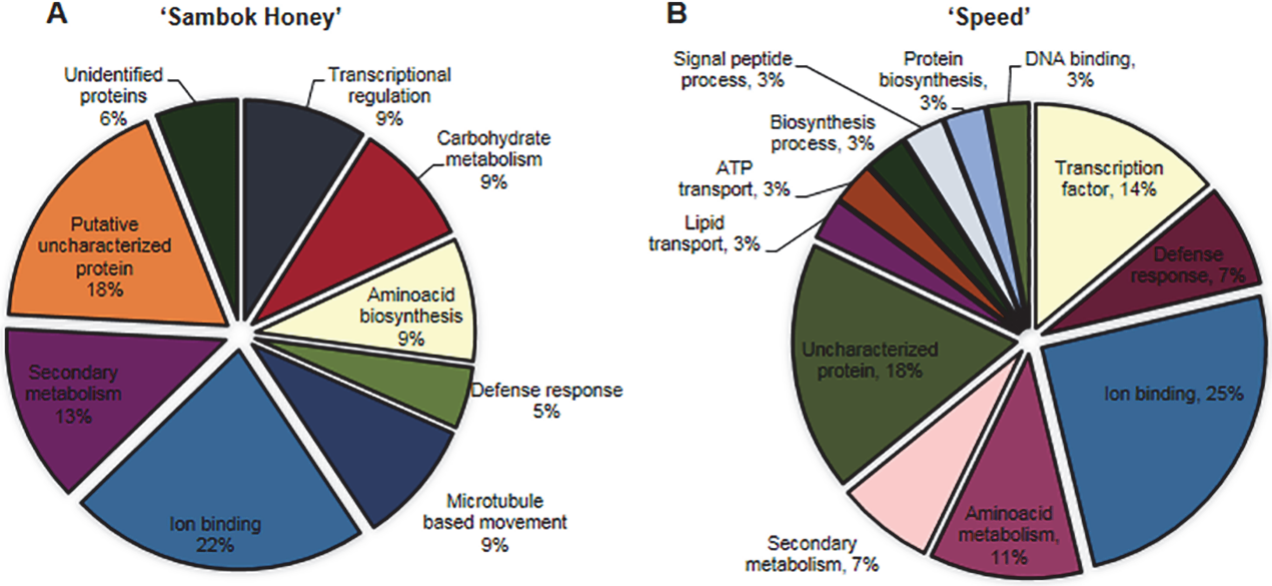
Functional classification of identified proteins from vascular connections (connected portions) in watermelon (*Citrullus vulgaris* Schrad.) ‘Sambok Honey’ and ‘Speed’ as the scion and bottle gourd (*Lagenaria siceraria* Stanld.) ‘RS Dongjanggun’ as rootstock seedlings grown under different photon flux densities (25, 50, 75 and 100 μmol m−2 s−1 PPFD). (A) ‘Sambok honey’ (B) ‘Speed’ as the scion and bottle gourd (*Lagenaria siceraria* Stanld.) ‘RS Dongjanggun’ as rootstock seedlings grown under different photon flux densities (25, 50, 75 and 100 μmol m^−2^ s^−1^ PPFD). The proteins identified were classified based on their putative biological functions.

All identified proteins were classified into different categories based on their biological function (www.geneontology.com) (Fig. 4A). Most of the proteins identified in the vascular connections of bottle gourd rootstock-grafted watermelon fell under the categories of ion binding (22%), amino acid biosynthesis (9%), defense response (5%), carbohydrate metabolism (9%), transcriptional regulation (9%), microtubule-based movement (9%), secondary metabolism (13%) and uncharacterized proteins (18%).

The vascular connection proteins identified in the ‘Speed’ cultivar were very similar to those of the ‘Sambok Honey’ cultivar. Twenty-seven proteins were revealed as differentially expressed among 25, 50, 75 and 100 μmol m^-2^ s^-1^. The up- and down-regulated proteins among the different light intensities are mostly related to ion binding and amino acid biosynthesis (Table 2) and were identified as P-glycoprotein 17 (spot 18), annexin (spot 20), magnesium-protoporphyrin (spot 22), ATP synthase subunit alpha (spot 23), large subunit GTPase (spot 26), phenylacetaldehyde synthase (spot 17) and thiamine biosynthesis family protein (spot 25). Two other important vascular connection proteins were found to be related to defense response: disease resistance family protein (spot 5) and catalase (spot 6).

**Table 2:** Proteins identified by MALDI-TOF/TOF-MS in watermelon ‘Speed’ as the scion and bottle gourd ‘RS Dongjanggun’ as rootstock.

Spot No.	NCBI accession number	Protein Name	Biological function	Plant Species	M*r* Value	Calculated p*I*/Exp p*I*	MASCOT Score	Sequence coverage (%)
1	M1D5Z5	Uncharacterized protein	-	*Solanum tuberosum*	9149	9.0/4.5	31	26
2	K7K6U0	Uncharacterized protein	-	*Glycine max*	27098	5.5/4.6	57	36
3	A8IZ15	Predicted protein	-	*Chlamydomonas reinhardtii*	32240	8.5/4.7	60	22
4	D3W146	Non-specific lipid-transfer	Lipid transport	*Phaseolus vulgaris*	12341	9.1/4.8	64	13
5	B9NHG2	Disease resistance family protein	Defense response	*Populus trichocarpa*	105560	5.1/4.4	74	38
6	A8J537	Catalase	Defense response	*Chlamydomonas reinhardtii*	56407	6.3/4.3	76	17
7	A9PK54	Transcription elongation factor SPT4 homolog	Transcription factor	*Populus trichocarpa*	13606	7.0/6.5	77	21
8	B9I128	DNA-binding bromodomain-containing family protein	DNA binding	*Populus trichocarpa*	63359	8.6/6.6	88	21
9	F2XX48	Glyceraldehyde-3-phosphate dehydrogenase	Oxidoreductase	*Litchi chinensis*	34063	6.1/6.1	76	35
10	J3RTS9	Glyceraldehyde-3-phosphate dehydrogenase	Oxidoreductase	*Cuscuta pentagona*	32382	6.7/4.5	82	32
11	D3W358	DNA-directed RNA polymerase	Transcription factor	*Platycladus orientalis*	18505	9.0/5.5	82	45
12	D7LLC7	UDP-glucoronosyl/UDP-glucosyl transferase family protein	Transferase activity	*Arabidopsis lyrata*	54068	6.0/6.0	80	16
13	Q93ZQ0	AT5g46390/MPL12_19	Serine-type peptide activity	*Arabidopsis thaliana*	23128	6.7/6.4	69	16
14	Q9FI43	Calcium-binding transporter-like protein	ATP transport	*Arabidopsis thaliana*	54984	8.9/5.5	64	31
15	U7DVE4	Uncharacterized protein	-	*Populus trichocarpa*	25103	9.2/4.5	73	35
16	D3W358	DNA-directed RNA polymerase	Transcription factor	*Platycladus orientalis*	18505	9.0/6.4	77	54
17	Q0ZS27	Phenylacetaldehyde synthase	Amino acid metabolism	*Rosa hybrid cultivar*	57077	6.7/5.7	63	19
18	D7LPT3	P-glycoprotein 17	ATP binding	*Arabidopsis lyrata*	137399	9.2/6.0	71	27
19	A8QMG1	Chalcone synthase	Biosynthesis process	*Dactylorhiza praetermissa*	29807	5.1/6.1	51	46
20	D3GC08	Annexin	Calcium ion binding	*Jatropha curcas*	36015	6.4/6.7	61	28
21	C1MLG4	S24-like peptidase	Signal peptide processing	*Micromonas pusilla*	19224	8.5/6.8	45	40
22	D3VMM9	Magnesium-protoporphyrin	Metal ion binding	*Arum italicum*	15698	9.5/6.9	41	26
23	Q9T7A2	ATP synthase subunit alpha	ATP binding	*Welwitschia mirabilis*	42700	9.2/6.9	71	31
24	U7DVE4	Uncharacterized protein	-	*Populus trichocarpa*	25103	9.2/7.0	57	27
25	D7LLB4	Thiamine biosynthesis family protein	Amino acid metabolism	*Arabidopsis lyrata*	72588	5.9/4.1	83	15
26	M8BGX0	Large subunit GTPase	GTP binding	*Aegilops tauschii*	67569	7.9/4.0	81	16
27	P36428-2	Isoform Cytoplasmic of Alanine—tRNA ligase	Protein biosynthesis	*Arabidopsis thaliana*	105961	8.8/4.1	74	25

The other proteins identified from vascular connections (speed cultivar) are associated with transcription and include transcription elongation factor SPT4 homolog (spot 7) and DNA-directed RNA polymerase (spots 11 and 16). Other proteins identified from ‘Speed’ under different light intensities are related to lipid transport (nonspecific lipid transfer, spot 4), DNA binding (DNA-binding bromodomain-containing family protein, spot 8), ATP transport (calcium-binding transporter-like protein, spot 14), biosynthesis process (chalcone synthase, spot 14), signal peptide processing (S24 like peptidase, spot 21), and a most important protein related to protein biosynthesis (isoform cytoplasmic of alanine—tRNA ligase, spot 27).

Proteins related to secondary metabolism were also revealed and identified as glyceraldehyde-3-phosphate dehydrogenase (spots 9 and 10), UDP-glucoronosyl/UDP-glucosyl transferase family protein (spot 12), and AT5g46390/MPL12_19 (spot 13). The remaining proteins were not identified and categorized as predicted protein (spot 3) and uncharacterized proteins (spots 1, 2 and 24).

All the proteins identified from ‘Speed’ were classified into 12 categories according to gene ontology (www.geneontology.com) (Fig. 4B). The identified proteins were grouped in the classifications of amino acid metabolism (11%), ion binding (25%), defense response (7%), transcription factor (14%), DNA binding (3%), protein synthesis (3%), signal peptide processing (3%), Biosynthesis process (3%), ATP transport (3%), lipid transport (3%), secondary metabolism (7%) and uncharacterized proteins (18%).

### Amino acid biosynthesis response proteins

Five proteins closely related to amino acid biosynthesis (see Tables 1 and 2, biological function) were identified by the proteomic analysis. The up-regulation of these proteins, particularly under 100 μmol m^-2^ s^-1^, in the vascular connections of grafting suggests that amino acid biosynthesis is promoted via the rootstock [4, 21] and that these proteins might be involved in the transport and accumulation of ions to the scion during the healing process [32–33]. Accordingly, the accumulation of enzymes involved in amino acid biosynthesis in the vascular portion of grafted watermelon will possibly depend on a strong ability to take up nutrient ions, such as nitrogen and iron, by the root system of bottle gourd. It was also observed that amino acid biosynthetic proteins were largely down-regulated under low light intensities (25, 50, and 70 μmol m^-2^ s^-1^), in contrast to the up-regulation under higher light intensity (100 μmol m^-2^ s^-1^). These results indicate that the biosynthesis of proteins related to amino acid metabolism is intensified for transport processes between the rootstock and scion under higher light intensity. Moreover, it was also revealed that 100 μmol m^-2^ s^-1^ light is the optimal condition for successful grafting.

### Defense response proteins

Defense response proteins play an important role in ameliorating the effects of several abiotic stresses [34–35]. It is also believed that proteins related to defense responses play a significant role in healing during grafting [6, 15]. The proteins related to defense mechanism group was earlier also observed in xylem [36] and phloem sap [37] depicting a greater contribution for normal translation and translocation of minerals in response to biotic or abiotic stress. Indeed the stress regulated genes was also observed in the transcriptomic analysis of phloem and xylem [38–39]. This indicates that proteins related to defense mechanisms is largely involved in cambial activities. Interestingly, our proteome data revealed proteins related to defense responses in the vascular connections (grafted portion) of rootstock and scion, such as disease resistance proteins (spot 7, Table 1, and spot 5, Table 2) and catalase (spot 6, Table 2). Disease resistance proteins are induced when grafted plants face attack by soil-borne pathogens, and catalase is primarily induced when plants are under abiotic stress [34–35]. Our results showed that defense response proteins were up-regulated in our rootstock-grafted scions. This result indicates that defense response proteins accompany a strong antioxidant mechanism in the vascular portion of a rootstock-grafted scion for successful grafting and tolerance to the mechanical injury produced during the grafting process. Based on our proteome study, we also observed that under 100 μmol m^-2^ s^-1^ light intensity, defense proteins were more highly up-regulated compared to the other light intensities, indicating that 100 μmol m^-2^ s^-1^ is the optimal light intensity for the healing process.

### Ion-binding response proteins

As ion-binding proteins play an important role in the vascular tissues of plants with regard to the transfer of ions [40–41], it is interesting to examine proteins related to ion binding between the two connected portions in rootstock-grafted scions. A greater number of proteins related to ion binding have been well identified in cambial activities previously in plants, which depicted that these proteins play an important role in translation and translocation of mineral in plants [42–43]. Indeed xylem ionic protein relations have been observed well to stress responses [43]. The proteomic analysis in our study revealed most of the proteins in the vascular connections of both scion cultivars are related to ion binding (see Tables 1 and 2, biological functions). The identification of these proteins revealed that proteins related to ions play key roles in the transfer of ions from the rootstock to scion during the healing process after grafting to achieve healthy growth. It was also observed that rootstock-grafted scions exhibit the up-regulation of ion-binding proteins under 100 μmol m^-2^ s^-1^ compared to the other experimental light intensities tested. These results indicate that ion-binding proteins might be a key factor for the healing process after grafting by helping the rootstock transfer ions to the scion. By up-regulating ion-binding proteins under 100 μmol m^-2^ s^-1^, our results also indicate that rootstock-grafted scions heal best at this light intensity.

### Transcription-responsive proteins

The proteomic analysis of the rootstock-grafted scion vascular proteome revealed a number of proteins related to transcription factors. Many studies of transcriptional activation have reported that transcription factors play a major role in stress response pathways [44–46]. Indeed a number of xylem and phloem transcriptomes from secondary tissues have identified genes related to transcription factors required for vascular cell differentiation and function [47–49]. Our proteomic analysis showed that proteins identified in the vascular connections of rootstock-grafted scions are related to transcription factors (see Table 1 and Table 2), indicating that transcription factors might play an enhanced role in the defense response produced by mechanical injury during grafting. Correspondingly, a separate groups of proteins related to defense responses have already been described above for their role in rootstock and scion vascular connections. The analysis of transcription factor proteins in vascular connections also suggested that these proteins might be involved in regulating primary meristems and cambial activities during graft union formation. Furthermore, most of the transcription-related proteins were up-regulated under 100 μmol m^-2^ s^-1^ and down-regulated under 25, 50 and 75 μmol m^-2^ s^-1^, which indicated that transcription-responsive proteins in the vascular connections of rootstock bottle gourd grafted with ‘Sambok Honey’ and ‘Speed’ watermelon might be optimally synthesized at 100 μmol m^-2^ s^-1^ light intensity for healthy growth and successful healing after grafting.

### Carbohydrate metabolism-related proteins

Carbohydrate metabolism related proteins in vascular cambium exudates long-distance signaling in plants which play an important role in plant growth and development [50–51]. The carbohydrates are indeed main components in plants for all metabolic activities such as glycolytic pathways [51]. The number of proteins belong to carbohydrate group have been identified in vascular sap of plants [52–53]. The present study revealed proteins related to carbohydrate metabolism in rootstock-grafted scions, including beta-galactosidase (spot 3, Table 1) and beta-amylase (spot 17, Table 1). These proteins help to provide plants with carbohydrates and energy during biotic or abiotic stresses and are best known for their expression during fruit development [54]. Therefore, the up-regulation of these proteins at 100 μmol m^-2^ s^-1^ indicates that they might support rootstock-grafted scions for the better development of fruits and mechanical shocks during and after grafting. Moreover, the identification of carbohydrate group proteins indicates a signaling exudation between rootstock and scion for better plant growth and development.

### Protein synthesis

Proteins synthesis plays an important role in the plant cell with regard to many physiological processes in response to unfavorable conditions [46]. The expression of isoform cytoplasmic of alanine-tRNA ligase (spot 27, Table 2), which plays a crucial role in protein synthesis, was observed to be up-regulated under 100 μmol m^-2^ s^-1^ in the vascular connections of rootstock-grafted scions. The up-regulation of this enzyme might enhance the translational process or enhance protein synthesis in the two grafted portions. These results indicate that rootstock bottle gourd grafted with ‘Sambok honey’ and ‘Speed’ might optimally synthesize proteins for several other metabolic processes at 100 μmol m^-2^ s^-1^.

### Comparison of differentially expressed proteins among different light intensities

**Fig 5:**
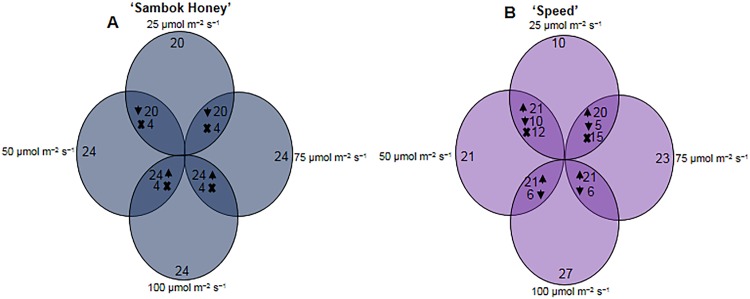
Comparative analysis of vascular connection proteome. A graphical representation of the number of differentially accumulated proteins in watermelon (*Citrullus vulgaris* Schrad.) ‘Sambok Honey’ and ‘Speed’ as the scion and bottle gourd (*Lagenaria siceraria* Stanld.) ‘RS Dongjanggun’ as rootstock seedlings grown under different photon flux densities (25, 50, 75 and 100 μmol m−2 s−1 PPFD). (A) ‘Sambok honey’ (B) ‘Speed’ as the scion and bottle gourd (*Lagenaria siceraria* Stanld.) ‘RS Dongjanggun’ as rootstock. Numbers correspond to the protein spots present in 2DE patterns under 25, 50, 75 and 100 μmol m^−2^ s^−1^. The *overlapped* portions with ‘X’ denote absence of proteins. *Upward* and *downward* arrows denote increased or decreased protein expression under combined treatments.

A comparison of the proteome maps of rootstock bottle gourd grafted with the watermelon cultivars ‘Sambok Honey’ and ‘Speed’ were compared under different light intensities (25, 50, 75 and 100 μmol m^-2^ s^-1^) and is described by the graphical diagram shown in Fig. 5. For ‘Sambok Honey’, 20 protein spots were down-regulated and 4 were absent when grown (healing period) at 25 μmol m^-2^ s^-1^ compared to the other light intensities (50, 75 and 100 μmol m^-2^ s^-1^) (Fig. 5A).

However, drastic differences between the four light intensities were observed for ‘Speed’ (Fig. 5B). Of 27 differentially expressed protein spots, only 9 were observed under 25 μmol m^-2^ s^-1^, whereas 21 were observed under 50 μmol m^-2^ s^-1^ and 24 under 75 μmol m^-2^ s^-1^. A comparison revealed that 14 proteins were up-regulated and 7 protein spots were down-regulated at 25 μmol m^-2^ s^-1^ compared to 50 μmol m^-2^ s^-1^. Of the 21 protein spots, 12 were absent at 25 μmol m^-2^ s^-1^ compared to 50 μmol m^-2^ s^-1^. A comparison revealed that of 24 protein spots, 20 were up-regulated, 5 down-regulated and 15 absent at 25 μmol m^-2^ s^-1^ compared to 75 μmol m^-2^ s^-1^. An interesting comparison was made between 25 and 100 μmol m^-2^ s^-1^; the proteomic maps showed that of 27 proteins spots, only 9 were present at 25 μmol m^-2^ s^-1^, though they were largely down-regulated.

The comparison of proteomic maps among the different light intensities was performed to reveal the optimal light intensity for healing after grafting and for tolerance to the mechanical injury suffered during the grafting process. Our results suggest that 100 μmol m^-2^ s^-1^ is an optimal light intensity for healing after grafting and for the synthesis of proteins related to amino acid biosynthesis, ion binding, defense response and transcription factors. A comparative analysis of the proteins related to multiprotein complexes also suggested that high light intensity can be optimal for the growth and development of plants [17].

### Vascular transport activity in rootstock-grafted scions

**Fig 6:**
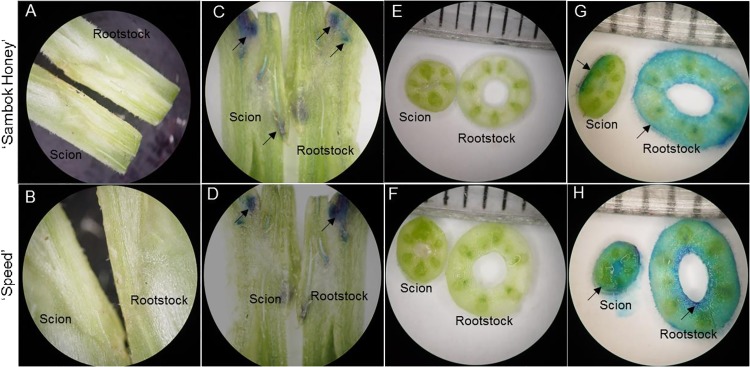
Visualization of water uptake and transport in watermelon (*Citrullus vulgaris* Schrad.) ‘Sambok Honey’ and ‘Speed’ as the scion and bottle gourd (*Lagenaria siceraria* Stanld.) ‘RS Dongjanggun’ as rootstock seedlings grown under different photon flux densities (25, 50, 75 and 100 μmol m−2 s−1 PPFD). The rootstock were submerged in absorbable flower dye blue for 10–20 min and both rootstock and scion were cut into transverse and longitudinal cross section and were observed under light microscope. The arrows in vascular tissues represent the vascular transport activity. (A-B) represents longitudinal section without flower dye blue; (C-D) represents longitudinal section with flower dye blue; (E-F) represents transverse section without flower dye; (G-H) represents transverse section with flower dye blue in rootstock and scion.

To check vascular transport activity between a rootstock and scion, a reliable staining ‘absorbable flower dye blue’ method was utilized in plants grown at 100 μmol m^-2^ s^-1^ (100 μmol m^-2^ s^-1^ was selected based on the physiological and proteomic data). The rootstock-grafted scions were dipped in staining solution for 10–20 min, and a longitudinal and transverse cross-section of both the rootstock and scion was analyzed for vascular transport activity. We observed blue coloration in the vascular tissues of both the bottle gourd rootstock and watermelon ‘Sambok Honey’ and ‘Speed’ (Fig. 6).

The vascular transport activity in the rootstock and scion was classified by staining, as previously observed in grafted sweet cherry [55] and *Arabidopsis*-grafted plants [56]. The stained areas in the vascular connections of the rootstock-grafted scions indicated the appropriate and effective connections of vessels and the functional transport of nutrients and other important minerals between the rootstock and scion.

## Conclusions

Our proteomic analysis of the vascular connections between bottle gourd as the rootstock, ‘Sambok Honey’ and ‘Speed’ watermelon cultivars as the scion revealed interesting proteins that might be involved in strong connections of two organisms during the healing process to provide tolerance against stress conditions. Moreover, a comparative analysis of the proteins also identified the optimal light intensity for successful grafting. The proteins analyzed in rootstock-grafted scions represent only a small portion of the vascular connection proteome, and many other proteins associated with xylem and phloem for transport processes still need to be identified. A deeper proteomic analysis along with a microarray analysis at the vascular connection level can disclose the mechanism by which two organisms share a single transport route of nutrients and minerals for successful grafting.

## Supporting Information

S1 Fig Diagrammatic representation for protein extraction and strategy for 2D gel electrophoresis. (TIFF)
