# Supplementary figures and images for: Proteomic Study Related to Vascular Connections in Watermelon Scions Grafted onto Bottle-Gourd Rootstock under Different Light Intensities
Source: PLoS One. 2015 Mar 19;10(3):e0120899. doi: 10.1371/journal.pone.0120899 (PMC4366178; doi:10.1371/journal.pone.0120899)

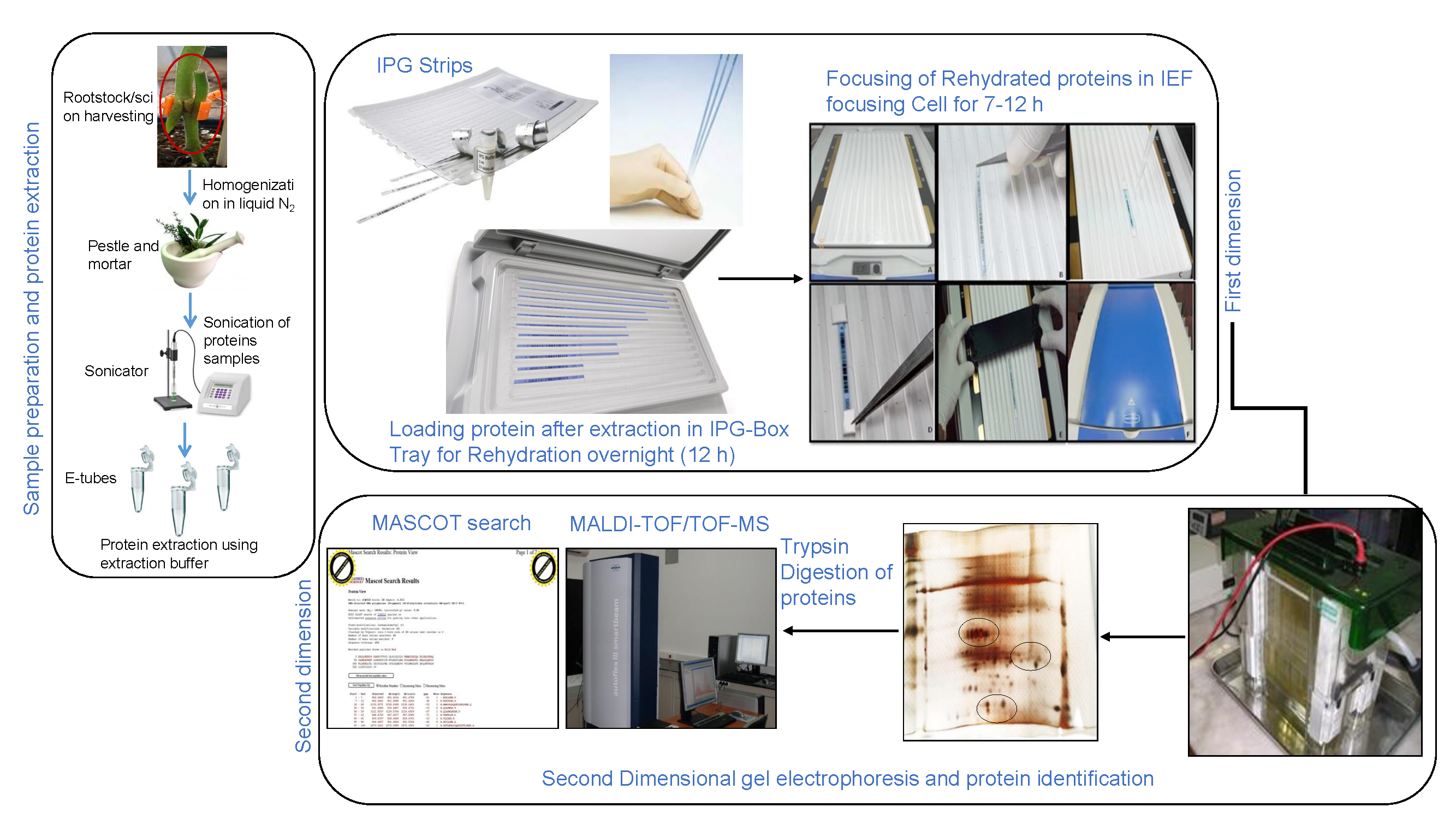

Supplement: S1 Fig — (TIFF) [file pone.0120899.s001.tiff]
